# Optimized Prediction Models from Fundus Imaging and Genetics for Late Age-Related Macular Degeneration

**DOI:** 10.3390/jpm11111127

**Published:** 2021-11-01

**Authors:** Arun Govindaiah, Abdul Baten, R. Theodore Smith, Siva Balasubramanian, Alauddin Bhuiyan

**Affiliations:** 1 iHealthscreen Inc., New York, NY 11418, USA; arun@ihealthscreen.org; 2 AgResearch, Palmerston North 4442, New Zealand; Abdul.Baten@agresearch.co.nz; 3 New York Eye and Ear Infirmary, New York, NY 10003, USA; rts1md@gmail.com; 4 Genetech, South San Francisco, CA 94080, USA; balasiva@gene.com

**Keywords:** macular degeneration, genetics, fundus imaging, deep learning

## Abstract

Age-related macular degeneration (AMD) is a leading cause of blindness in the developed world. In this study, we compare the performance of retinal fundus images and genetic-information-based machine learning models for the prediction of late AMD. Using data from the Age-related Eye Disease Study, we built machine learning models with various combinations of genetic, socio-demographic/clinical, and retinal image data to predict late AMD using its severity and category in a single visit, in 2, 5, and 10 years. We compared their performance in sensitivity, specificity, accuracy, and unweighted kappa. The 2-year model based on retinal image and socio-demographic (S-D) parameters achieved a sensitivity of 91.34%, specificity of 84.49% while the same for genetic and S-D-parameters-based model was 79.79% and 66.84%. For the 5-year model, the retinal image and S-D-parameters-based model also outperformed the genetic and S-D parameters-based model. The two 10-year models achieved similar sensitivities of 74.24% and 75.79%, respectively, but the retinal image and S-D-parameters-based model was otherwise superior. The retinal-image-based models were not further improved by adding genetic data. Retinal imaging and S-D data can build an excellent machine learning predictor of developing late AMD over 2–5 years; the retinal imaging model appears to be the preferred prognostic tool for efficient patient management.

## 1. Introduction

Age-related macular degeneration (AMD) is the leading cause of visual disability in the developed world and a leading cause globally [1]. Approximately 11 million individuals are affected with late AMD in the United States of America (USA) alone, with a global prevalence of 170 million in 2015 [2,3]. Aging is the most significant risk factor. The prevalence of late AMD in the USA is expected to increase to 22 million by the year 2050, while the global prevalence is expected to increase to 288 million by the year 2040 [2].

AMD has been associated with many genetic and environmental risk factors and their interactions [4]. Early detection and referral to an ophthalmologist could enable management options such as photobiomodulation [5], laser intervention [6], or other strategies that may arise in the future.

Our literature review found three publications on the prediction of late AMD progression. A model proposed by Bhuiyan et al. [7], color fundus image and socio-demographic (S-D)-data-based prediction models for 2-year, 5-year and 10-year incidence achieved up to 86.4% accuracy. Yan et al. [8] found 85% accuracy for their 7-year incidence prediction model. In one of our previous papers [9], we proposed a screening method which showed over 92.5% accuracy. There are also AMD screening approaches quantifying drusen using traditional non-deep-learning-based approaches [10]. In a research paper by Wu et al. [11], a color fundus image and optical coherence tomography (OCT) image-based prediction achieved an AUC of 0.88 for 3-year incidence. The genetic loci and their association with late AMD progression has been summarized in [12] with providing the odds ratio (OR) (maximum OR 8.59 for ARMS2) for individual locus. Our study did not find any model that is based on genetic information only. A review of various methods using traditional approaches was given by Kanagasingam et al. [13]

The models we test and optimize are machine learning (ML)-based models built on retinal images, genetic and socio-demographic (S-D) parameters, and their combinations. We also compare these models with our recently developed late AMD prediction model using retinal fundus photos and (S-D) parameters [7], which predicts an individual at risk of developing late AMD within 2 years, 5 years, and 10 years. We have used the combinations of the input variables to study the best parameters for the prediction of late AMD and validate the best method to predict the disease at an early stage and help the prevention of blindness.

## 2. Materials and Methods

The methods section is organized as follows. First, the dataset is described briefly along with the acquisition of the dataset. Second, the different types of data used in the study are described, namely, genetic data, socio-demographic and health data, and retinal image data. Third, we describe the various models we built for comparison, i.e., the various combinations of the different types of data used to build the models. Lastly, we describe the statistical measures used in this comparison study.

Briefly, the main points of the methods are as follows:

1. For building AMD prediction models, we used retrospective dataset made available by the AREDS study [14]. It consists of data from 4146 participants who enrolled in the study and were monitored for over 13 years during the course of the study.
2. We wanted to analyze the best and the most useful predictors of AMD disease, so we built and compared statistical and machine learning models with a variety of different combinations of data types (retinal, genetic, and medical data).
3. In total, there were 566 participants chosen from the AREDS study based on the availability of all the retinal, medical, and genetic data.
4. In the case of retinal image data, we already had deep-learning-based classifiers that were built using over 100,000 images from the AREDS dataset, not including the data from 566 participants used in this comparative study. The dataset was split into separate training, validation, and testing sets in the ratio of 60:20:20, respectively. The automated grading by these classifiers was used as parameters in building further machine learning models along with the other genetic and medical (and socio-demographic) parameters.
5. Over the course of the AREDS study, conversions to late AMD are recorded in yearly visits. To build prediction models, we prepared datasets which included participants whose eyes converted to late AMD in 2, 5, and 10 years.
6. For each of these durations, we separately built models using combinations of retinal, socio-demographic, and genetic data.
7. Lastly, we analyzed the best predictors for each of the duration (2, 5, and 10 years) and proposed the best models.

### The Age-Related Eye Disease Study (AREDS)

The AREDS is a study on late AMD sponsored by the National Eye Institute [14]. The AREDS participants were 55 to 80 years old at enrollment, and they had to be free of any illness or condition that would make a long-term follow-up or compliance with study medications unlikely. Based on fundus photographs graded by a central reading center, the best-corrected visual acuity, and ophthalmologic evaluations, 4753 participants were enrolled in one of several AMD categories, including persons with no AMD. Broadly, the information collected in the follow-up visits were socio-demographic and clinical data (e.g., blood pressure or diabetes), genetic data, and retinal fundus image data. In our study, the number of subjects was based on the availability of genetic data in the AREDS dataset, as explained in the following subsections.

## 3. Data Acquisition

### 3.1. Genotype Data

The AREDS dataset contains genetic data (SNPs) for 568 subjects, 381 cases of advanced AMD (exudative (246), atrophic (184), or both (51)) and 187 normal cases [15]. We restricted our study to these AREDS subjects for the 2-year models. For the 5- and 10-years models, there were 566 participants with data available: 183 with atrophic AMD, 246 with exudative AMD, 50 with both forms, and 187 normal cases.

The current literature suggests that risk alleles of genes ARMS2, CFH, and SNPs in C2/CFB rs641153, C3 rs2230199, C2/CFB rs4151667 are linked with late AMD (Table A1 and Table A2 in the Appendix A), and that high risk is attributable to the one SNP of ARMS2 and the five SNPs of CFH (Table A2) [15,16,17,18,19] that we have considered in this study. These are CFH SNPs-rs380390, rs572515, rs800292, rs1329428, rs10801575, and ARMS2-rs10490924. The genotyping was done by AREDS using the Illumina HumanOmni2.5 platform [20]. The risk ratios of these SNPs for late AMD outcomes, with associated *p* values, are shown in Table A1.

### 3.2. Socio-Demographic Data

The AREDS subjects were randomly assigned to the vitamins and mineral supplements and placebo groups [14]. Socio-demographic data, along with physiological data, were collected every six months from the participants, and altogether 13 years of follow-up data are available in the dataset. For this study, we considered socio-demographic, physiological, and clinical data taken periodically during that time. They include gender, age, smoking status, diabetes status, body mass index (BMI), blood pressure, sunlight exposure, and visual acuity. From this longitudinal data, for those subjects with incident late AMD, study data were taken from one visit approximately within 2 years of the disease diagnosis for building the 2-year risk prediction model, and the data were taken similarly for the 5-year model. For subjects without incident late AMD within 2 years (similarly, 5 years and 10 years for the 5-year and 10-year models), data from one random visit during the longitudinal data were taken. Therefore, all subjects with and without incident late AMD had exactly one row of data in the final dataset, which was used for the analysis and model building.

### 3.3. Retinal Image Data

AREDS has defined a 9-point scale based on the retinal image for the risk of late AMD progression [21]. The risk of patients converting to late AMD ranges from about 2% in level 1 to about 50% in level 9 on that scale. To include the images that do not fall in the 9-point scale, i.e., the images that already show progression, the scale was extended to add 3 more levels to define advanced AMD for grading all the images. The 10, 11, and 12 levels indicate late dry AMD only, late wet AMD only, and both dry and wet late AMD. In total, we used twelve levels to develop AMD prediction models. Along with AMD severity, AREDS data contain graded information about the AMD category [14]. Four categories are defined, namely, 1 to 4, based on the presence and extent of drusen and other AMD pathologies. Category 1 is referred to as normal, 2 as early, 3 as intermediate, and 4 as advanced or late AMD. A deep-learning-based automated classifier pre-classified the images into these AREDS defined categories automatically. This retinal-image-based grading algorithm can be summarized as follows.

The deep-learning-based automated AMD category and severity classifiers made use of over 80,000 images from the AREDS dataset, which had been graded for AMD severity and category by the AREDS grading center. Extra care was taken to ensure that no participant whose images are used in these automated models also appears in the AMD prediction model that was built solely on those participants who have genetic data, as explained in the previous sections.

### 3.4. Data Categories

The input variables are a combination of continuous variables, binary variables, and categorical variables. The clinical data and socio-demographic data are continuous and binary, whereas image parameters are categorical (AMD category and severity), and the SNPs are binary variables. Keeping the reference allele frequency as base, the two allele frequencies are taken as two separate input variables for every SNP.

### 3.5. Late AMD Prediction

Socio-demographic data, clinical data, fundus image data, and genetic data were used to build the models for late AMD prediction. Six different machine learning models were implemented and compared in this study with these sets of input variables (Table 2):

1. Socio-demographic/Clinical data.
2. Genetic data.
3. Automated AMD grades from retinal images.
4. Socio-demographic/clinical data and genetic data.
5. Socio-demographic/clinical data and retinal image data.
6. All the input variables (1–5).

### 3.6. Ensemble Approach to Model Building

Our deep learning (DL)-based classifier [22] is an ensemble of DL AMD classifiers developed earlier that determine from a retinal image the stage of AMD present (no AMD, early AMD, intermediate AMD, and late AMD) [23]. The classifiers are five networks of different image input sizes of “Inception-V3” proposed by Szegedy et al. [24], “Inception-Resnet-V2” proposed by Szegedy et al. [25], and “Xception” proposed by Chollet [26]. The ensemble approach was found to be the best performing experimentally compared to any individual classifier.

**Figure 1:**
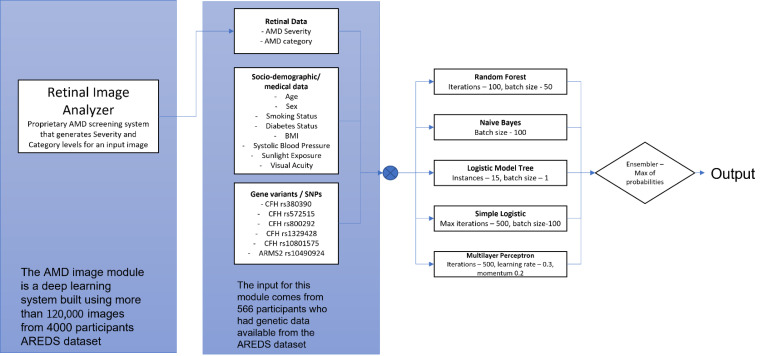
The overall structure of the late AMD prediction model.

Another ensemble deep learning classifier [27] was used to assign probabilities that an image falls within each of the 12 AREDS classes; specifically, for images without late AMD, the probabilities of falling within the first 9 classes. This system consists of an ensemble of six neural networks, each differing from the other with respect to the combination of input image size and the network architecture. The six networks are: Xception network with input size 499 × 499, Inception-Resnet-V2 network with size 399 × 399, Xception network with size 299 × 299, Inception-V3 network with input size 599 × 599, Inception-V3 with input size 399 × 399, and NasNet network (proposed by Zoph et al. [28]) with input size 399 × 399. Each network is trained to give an array of 12 probabilities, one for each class, whose values are then combined to give the most probable AMD severity level. The AMD stage and severity level are then the retinal image inputs to the 6 machine learning (ML) AMD prediction models just listed. The complete set of input variables (Figure 1, left column) are normalized and scaled.

### 3.7. Model Optimization

Five separate machine learning (ML) and statistical algorithms (Bayesian modeling [29], logistic regression modeling [30], decision trees [31], random forest [32], and logistic model tree [33]) were ensembled in each of the 6 models to provide optimum prediction probabilities. Random forest (100 iterations, batch size of 50, and unlimited maximum depth), naïve Bayes (batch size of 100), logistic model tree (15 instances, 1 boosting iteration, and batch size of 1), simple logistic (max boosting iterations set at 500, heuristic stop at 50, and batch size of 100), and multilayer perceptron (with 500 iterations, a learning rate of 0.3, and momentum of 0.2). Due to the relatively small size of the dataset, we used 10-fold cross-validation for assessing each model’s performance. Each prediction model had two separate subsystems, for predicting late dry AMD and late wet AMD, respectively. Both subsystems operated with the same procedure and input variables as the parent system, with their own target outcomes

The deep learning models were implemented using the Keras framework with the TensorFlow backend. Pandas and lifelines libraries were used for the ML/statistical models. The training data are available in the public domain upon request from dbGaP that holds the AREDS image and genotype datasets. The code and trained models are available upon request.

### 3.8. Statistical Measures

The final AMD prediction system contains several sub-methods and algorithms which were evaluated separately and as a whole. Retinal-image-based classifiers that classify the images into 4 categories and 12 levels were evaluated using categorical accuracy, loss, and quadratic weighted kappa. More on this was published in our detailed research publication on stratifying AMD automatically [23]. The individual machine learning models were evaluated using accuracy measures based on the two classes—AMD or no AMD. The final classifier, which is an ensembled model, was evaluated based on several clinically relevant measures such as sensitivity, specificity, accuracy with AMD as positive, and no AMD as negative. The model was also evaluated by plotting the receiver operating curve. The area under the curve was also calculated for the final models. Kappa scores were calculated for each of the models and are used for comparison. For all the measures, 95% confidence intervals were calculated and presented for comparison.

## 4. Results

Table 1 shows the detailed results of the two-year prediction model on sensitivity, specificity, and accuracy measures. The table also shows the results for the two subsystems with the same measures. The 2-year AMD prediction system achieves a high accuracy of 89.61% (95% CI—86.81% to 92.00%) with a sensitivity 92.13% (88.95% to 94.62%), a specificity of 84.49% (78.49% to 89.36%) when all the input variables are used. When only data from socio-demographic and clinical variables are taken, the model achieves an accuracy of 72.01% (68.12% to 75.66%).

Table 2 details the results of the 5-year AMD prediction system that achieves an accuracy of 87.21% (84.34% to 91.29%) with a sensitivity of 90.11% (87.05% to 93.32%), a specificity of 83.45% (76.49% to 88.26%) when all the input variables are used. When only data from socio-demographic and clinical variables are taken, the model achieves an accuracy of 68.07% (63.12% to 74.06%).

**Figure 2:**
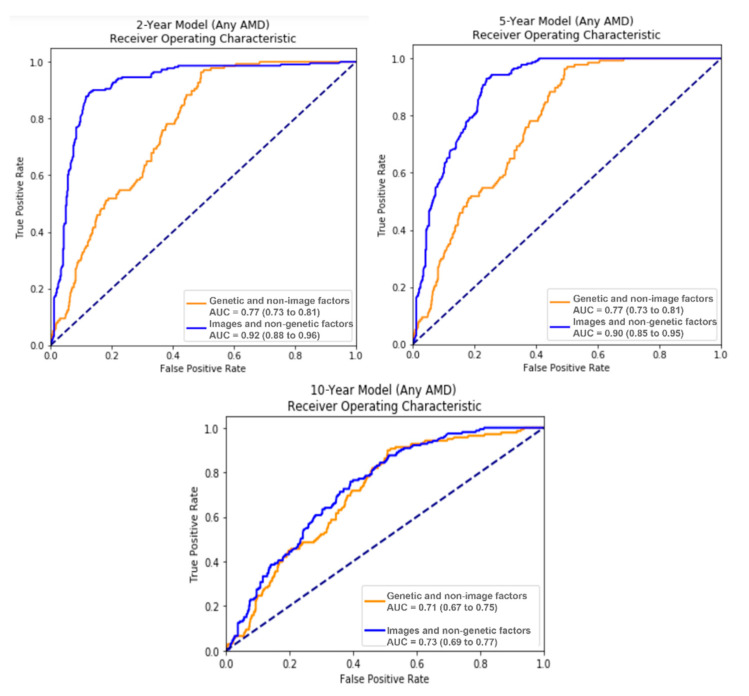
The ROC curves showing the performance of 2-year, 5-year, and 10-year prediction models.

Figure 2 shows the ROC curves from the 2-year and 5-year late AMD prediction models.

Most importantly, retinal image input variables by themselves perform very well compared to the all-input 2-year model, achieving an accuracy of 87.50% (84.50% to 90.11%) with a sensitivity of 89.24% (85.69% to 92.17%), a specificity of 83.96% (77.90% to 88.91%). When the subsystems are considered individually, the performance decreases comparatively across the board but still maintains decent measures. The accuracy of the all-input model for dry AMD model is 73.32% (68.51% to 77.75%) with a sensitivity of 75.00% (68.10% to 81.08%), a specificity of 71.66% (64.62% to 77.99%). The accuracy of the all-input model for wet AMD is 72.64% (68.19% to 76.78%) with a sensitivity of 72.98% (67.00% to 78.41%) and a specificity of 72.19% (65.18% to 78.48%).

Like the 2-year model, retinal image input variables in the 5-year model by themselves perform very well, comparable to the all-input model, achieving an accuracy of 86.2% (82.9% to 88.56%) with a sensitivity of 87.77% (84.61% to 91.56%), a specificity of 82.67% (76.31% to 87.91%). The accuracy of the all-input model for dry AMD model is 72.69% (67.01% to 77.43%) with a sensitivity of 74.51% (67.10% to 80.53%), a specificity of 70.98% (64.03% to 77.12%). The accuracy of the all-input model for wet AMD is 72.23% (67.99% to 76.71%) with a sensitivity of 72.21% (66.3% to 78.41%) and a specificity of 72.03% (65.18% to 77.48%). Similar results about the 10-year model can be found in Table 3.

## 5. Discussion and Conclusions

In this paper, we have provided a novel comparative study between image- and genetic-data-based late AMD prediction models. The results clearly show that the best models of AMD incidence within two years are based on retinal images. The addition of socio-demographic, clinical, and genetic data improved their overall performance, but were inferior predictors by themselves of late AMD incidence in two years and five years. The reason may be that the early signs of late AMD such as drusen or reticular pseudodrusen appear in retinal imaging. Predictive models could improve the management of higher risk patients in several ways: greater attention to modifiable risk factors such as body mass index (BMI), smoking, diet, and blood lipid levels [34]; increased motivation for decreasing these risks; increased motivation for both physician and patient for more frequent exams. Factors that cannot be modified at present include genotype at a given risk locus, sex, ethnicity, and age [34]. However, there is promising research into the early treatment of AMD in addition to oral AREDS supplements [35].

Although the genotype and phenotype were studied earlier to find the association with retinal disease, this paper addresses the challenge of predicting late AMD and shows a comparison between genetic and image-based late AMD prediction. In another similar model [8] a genetic and image-based AI model was proposed for predicting late AMD. However, our analysis showed that genetic information is more useful in addition to traditional risk factors in long-term scenarios when baseline imaging does not capture pathologic precursors of late AMD. However, for the 2-year and 5-year prediction of late AMD, imaging is much superior, sufficient, and highly accurate.

For this study, we used many different types of variables which may be hard to obtain in another similar study for external validation. However, we have tested a retinal-image-based model on an AMD study dataset called “NAT-2” [36]. This dataset contains images from 300 participants monitored for 3 years. We used expert graders to assess the referability of the images and tested it against our AMD screening system. The results were more than promising. The system correctly classified 82 out of 89 as non-referable for AMD (specificity 92.13; 95% CI 84.46% to 96.78%) and correctly classified 175 out of 199 as referable for AMD (sensitivity 87.94; 95% CI, 82.59% to 92.12%). The rest of the images were deemed ungradable.

For short-term late AMD prediction, genetic information alone was inferior to the high predictive value of baseline imaging severity of AMD. However, for 10-year late AMD prediction, results were similar: the genetics-based information achieved 68.2% accuracy with 75% sensitivity and 51.6% specificity, while the retinal-image-based model achieved 72.9% accuracy with 73.8% sensitivity and 72.7% specificity. Thus, image-based models are superior for shorter terms (2-year and 5-year), but not longer.

The models performed very well in clinically relevant measures, indicating that good late AMD prediction systems are within reach and ready for testing in real-world, prospective scenarios. We also found that these systems can predict the type of advanced AMD (dry or wet) with acceptable accuracy, but they could surely be improved by including spectral-domain optical coherent tomography (SD-OCT).

Our comparative study contrasts with another AI model that uses fundus images primarily for automating the standard AMD severity scores in general use by ophthalmologists. The authors of that proposed AI model did not include other risk factors such as age, smoking status, or AMD risk genetic variants. We believe that the prediction of late AMD progression will be more useful than severity score for patient management.

We anticipate that with an image-based prediction system, we can diagnose AMD early and can help to screen AMD fast and in a cost-effective way, which will lead to a large-scale screening through primary care settings. A further prospective study utilizing primary care settings can confirm the suitability of the screening. This is an essential approach that we screen through primary care settings, as when most subjects show up, it is late, and the only option is to stop the degeneration. Thus, if implemented, a screening in primary care settings, where most people visit regularly, will overcome the problem of not showing up at ophthalmic clinics timely. The individuals at risk of late AMD will be referred to an ophthalmologist who can make a further diagnosis and start preventative measures (e.g., AREDS supplement) or necessary treatment. This early intervention will eventually help the prevention of late AMD and unnecessary blindness.

We conclude that late AMD prediction by imaging only, including the dry and wet forms, is possible without genome sequencing for shorter time periods (2 to 5 years), but not significantly longer.

## Figures and Tables

**Table 1:** The performance comparison of the models with different inputs for predicting 2-year risk of developing “any AMD” (Dry or Wet AMD), dry AMD, and wet AMD. The measures such as sensitivity, specificity, accuracy, and kappa along with their 95% confidence intervals are given. Top accuracies are highlighted.

Input Variables	Socio-Demographic/Medical Data	Genetic Data Only	Socio-Demographic/Medical/Genetic Data	Retinal Images Data Only	Retinal Images/Socio-Demographic/Medical Data	All Input Variables
Sensitivity (any AMD)	77.69% (73.17% to 81.77%)	75.07% (70.41% to 79.33%)	79.79% (75.40% to 83.71%)	89.24% (85.69% to 92.17%)	91.34% (88.05% to 93.96%)	92.13% (88.95% to 94.62%)
Specificity (any AMD)	60.43% (53.03% to 67.49%)	51.34% (43.93% to 58.70%)	66.84% (59.60% to 73.54%)	83.96% (77.90% to 88.91%)	84.49% (78.49% to 89.36%)	84.49% (78.49% to 89.36%)
Accuracy (any AMD)	72.01% (68.12% to 75.66%)	67.25% (63.22% to 71.10%)	75.53% (71.78% to 79.01%)	87.50% (84.50% to 90.11%)	89.08% (86.23% to 91.53%)	89.61% (86.81% to 92.00%)
Unweighted kappa (any AMD)	0.38 (0.29 to 0.46)	0.26 (0.18 to 0.35)	0.46 (0.38 to 0.54)	0.72 (0.66 to 0.78)	0.75 (0.70 to 0.81)	0.77 (0.71 to 0.82)
Area under ROC (any AMD)	0.76 (0.72 to 0.80)	0.69 (0.65 to 0.74)	0.77 (0.73 to 0.81)	0.90 (0.86 to 0.94)	0.92 (0.88 to 0.96)	0.92 (0.88 to 0.96)
Sensitivity (dry AMD)	72.28% (65.22% to 78.61%)	71.20% (64.07% to 77.62%)	72.28% (65.22% to 78.61%)	74.46% (67.52% to 80.59%)	74.46% (67.52% to 80.59%)	75.00% (68.10% to 81.08%)
Specificity (dry AMD)	65.24% (57.95% to 72.04%)	52.41% (44.99% to 59.74%)	67.38% (60.16% to 74.04%)	70.59% (63.50% to 77.01%)	70.59% (63.50% to 77.01%)	71.66% (64.62% to 77.99%)
Accuracy (dry AMD)	68.73% (63.75% to 73.42%)	61.73% (56.57% to 66.69%)	69.81% (64.86% to 74.44%)	72.51% (67.66% to 76.99%)	72.51% (67.66% to 76.99%)	73.32% (68.51% to 77.75%)
Unweighted kappa (dry AMD)	0.38 (0.28 to 0.47)	0.24 (0.14 to 0.33)	0.40 (0.30 to 0.49)	0.45 (0.36 to 0.54)	0.45 (0.36 to 0.54)	0.47 (0.38 to 0.56)
Area under ROC (dry AMD)	0.72 (0.67 to 0.76)	0.67 (0.63 to 0.71)	0.72 (0.68 to 0.76)	0.75 (0.71 to 0.78)	0.75 (0.71 to 0.78)	0.78 (0.74 to 0.82)
Sensitivity (wet AMD)	70.97% (64.89% to 76.54%)	70.16% (64.05% to 75.79%)	71.37% (65.31% to 76.91%)	72.58% (66.58% to 78.03%)	72.98% (67.00% to 78.41%)	72.98% (67.00% to 78.41%)
Specificity (wet AMD)	64.71% (57.40% to 71.54%)	53.48% (46.05% to 60.79%)	67.91% (60.71% to 74.54%)	72.19% (65.18% to 78.48%)	71.66% (64.62% to 77.99%)	72.19% (65.18% to 78.48%)
Accuracy (wet AMD)	68.28% (63.67% to 72.63%)	62.99% (58.26% to 67.54%)	69.89% (65.33% to 74.16%)	72.41% (67.96% to 76.56%)	72.41% (67.96% to 76.56%)	72.64% (68.19% to 76.78%)
Unweighted kappa (wet AMD)	0.36 (0.27 to 0.44)	0.24 (0.15 to 0.33)	0.39 (0.30 to 0.48)	0.44 (0.36 to 0.53)	0.45 (0.36 to 0.53)	0.45 (0.36 to 0.53)
Area under ROC (wet AMD)	0.71 (0.67 to 0.75)	0.66 (0.62 to 0.70)	0.73 (0.69 to 0.77)	0.76 (0.72 to 0.80)	0.76 (0.72 to 0.80)	0.77 (0.73 to 0.81)

**Table 2:** The performance comparison of the models with different inputs for predicting 5-year risk of developing ‘any AMD’ (Dry or Wet AMD), dry AMD and wet AMD. The measures such as the sensitivity, specificity, accuracy and kappa along with their 95% confidence intervals are given.

Input Variables	Socio-Demographic/Medical Data	Genetic Data Only	Socio-Demographic/Medical/Genetic Data	Retinal Images Data Only	Retinal Images/Socio-Demographic/Medical Data	All Input Variables
Sensitivity (any AMD)	75.23% (72.78% to 80.01%)	75.00% (70.00% to 79.99%)	79.79% (75.40% to 83.71%)	87.77% (84.61% to 91.56%)	88.24% (85.4% to 91.91%)	90.11% (87.05% to 93.32%)
Specificity (any AMD)	60.01% (56.0% to 65.72%)	51.55% (43.99% to 59.67%)	66.84% (59.60% to 73.54%)	82.67% (76.31% to 87.91%)	82.49% (76.45% to 87.11%)	83.45% (76.49% to 88.26%)
Accuracy (any AMD)	68.07% (63.12% to 74.06%)	68.21% (63.02% to 71.56%)	75.53% (71.78% to 79.01%)	86.2% (82.9% to 88.56%)	86.58% (84.20% to 90.13%)	87.21% (84.34% to 91.29%)
Unweighted kappa (any AMD)	0.34 (0.26 to 0.43)	0.27 (0.18 to 0.36)	0.46 (0.38 to 0.54)	0.72 (0.66 to 0.78)	0.75 (0.70 to 0.80)	0.76 (0.70 to 0.81)
Area under ROC (any AMD)	0.74 (0.70 to 0.78)	0.70 (0.65 to 0.75)	0.77 (0.73 to 0.81)	0.88 (0.84 to 0.93)	0.90 (0.85 to 0.95)	0.90 (0.86 to 0.95)
Sensitivity (dry AMD)	63.56% (57.34% to 70.02%)	70.01% (63.29% to 74.65%)	71.19% (65.02% to 77.74%)	73.31% (67.02% to 79.51%)	73.46% (66.22% to 79.59%)	74.51% (67.10% to 80.53%)
Specificity (dry AMD)	62.34% (56.95% to 70.11%)	52.45% (44.99% to 59.74%)	66.38% (60.16% to 74.04%)	70.59% (63.50% to 77.01%)	70.59% (63.50% to 77.01%)	70.98% (64.03% to 77.12%)
Accuracy (dry AMD)	62.73% (57.75% to 70.21%)	62.22% (56.57% to 66.69%)	69.81% (64.86% to 74.44%)	72.06% (67.66% to 76.99%)	72.15% (67.36% to 76.99%)	72.69% (67.01% to 77.43%)
Unweighted kappa (dry AMD)	0.34 (0.26 to 0.44)	0.25 (0.14 to 0.33)	0.41 (0.31 to 0.50)	0.45 (0.36 to 0.54)	0.45 (0.36 to 0.54)	0.46 (0.37 to 0.56)
Area under ROC (dry AMD)	0.67 (0.63 to 0.71)	0.67 (0.63 to 0.71)	0.71 (0.68 to 0.76)	0.72 (0.68 to 0.76)	0.74 (0.70 to 0.78)	0.75 (0.71 to 0.80)
Sensitivity (wet AMD)	60.67% (54.89% to 66.24%)	71.06% (66.02% to 76.79%)	70.21% (65.01% to 74.91%)	71.52% (67.68% to 76.03%)	71.03% (67.00% to 76.41%)	72.21% (66.3% to 78.41%)
Specificity (wet AMD)	63.76% (56.40% to 69.54%)	54.18% (47.05% to 61.29%)	66.36% (61.71% to 74.99%)	73.19% (65.18% to 78.48%)	72.66% (64.62% to 77.99%)	72.03% (65.18% to 77.48%)
Accuracy (wet AMD)	62.28% (58.67% to 66.63%)	63.05% (58.39% to 67.59%)	70.03% (65.89% to 74.77%)	71.41% (67.02% to 75.96%)	71.01% (67.86% to 75.56%)	72.23% (67.99% to 76.71%)
Unweighted kappa (wet AMD)	0.32 (0.27 to 0.39)	0.25 (0.15 to 0.32)	0.39 (0.30 to 0.48)	0.44 (0.36 to 0.53)	0.44 (0.36 to 0.53)	0.44 (0.36 to 0.53)
Area under ROC (wet AMD)	0.69 (0.65 to 0.74)	0.67 (0.63 to 0.71)	0.73 (0.69 to 0.77)	0.75 (0.72 to 0.80)	0.75 (0.72 to 0.80)	0.75 (0.72 to 0.80)

**Table 3:** The performance comparison of the models with different inputs for predicting 10-year risk of developing “any AMD” (dry or wet AMD), dry AMD and wet AMD. The measures such as the sensitivity, specificity, accuracy, and kappa along with their 95% confidence intervals are given.

Input Variables	Socio-Demographic/Medical Data	Genetic Data Only	Socio-Demographic/Medical/Genetic Data	Retinal Images Data Only	Retinal Images/Socio-Demographic/Medical Data	All Input Variables
Sensitivity (any AMD)	68.23% (62.78% to 80.01%)	75.00% (70.00% to 79.99%)	75.79% (70.40% to 83.71%)	73.77% (64.61% to 81.56%)	74.24% (65.4% to 81.91%)	76.11% (67.05% to 83.32%)
Specificity (any AMD)	56.01% (56.0% to 65.72%)	51.55% (43.99% to 59.67%)	66.84% (59.60% to 73.54%)	72.67% (66.31% to 77.91%)	72.49% (66.45% to 77.11%)	73.45% (66.49% to 78.26%)
Accuracy (any AMD)	64.07% (63.12% to 74.06%)	68.21% (63.02% to 71.56%)	70.53% (71.78% to 79.01%)	72.9% (70.9% to 77.56%)	73.58% (64.20% to 80.13%)	75.21% (64.34% to 81.29%)
Unweighted kappa (any AMD)	0.33 (0.26 to 0.43)	0.27 (0.18 to 0.36)	0.46 (0.38 to 0.54)	0.53 (0.46 to 0.68)	0.55 (0.40 to 0.70)	0.55 (0.40 to 0.61)
Area under ROC (any AMD)	0.71 (0.62 to 0.78)	0.70 (0.65 to 0.75)	0.71 (0.63 to 0.81)	0.73 (0.84 to 0.93)	0.73 (0.65 to 0.85)	0.75 (0.66 to 0.85)
Sensitivity (dry AMD)	61.56% (57.34% to 70.02%)	70.01% (63.29% to 74.65%)	71.19% (65.02% to 77.74%)	65.31% (57.02% to 69.51%)	68.46% (66.22% to 79.59%)	71.99% (67.10% to 80.53%)
Specificity (dry AMD)	60.34% (56.95% to 70.11%)	52.45% (44.99% to 59.74%)	65.38% (60.16% to 74.04%)	66.59% (63.50% to 71.01%)	67.59% (53.50% to 67.01%)	69.98% (64.03% to 77.12%)
Accuracy (dry AMD)	60.73% (57.75% to 70.21%)	62.22% (56.57% to 66.69%)	68.81% (54.86% to 74.44%)	65.66% (57.66% to 76.99%)	65.95% (57.36% to 76.99%)	70.69% (67.01% to 77.43%)
Unweighted kappa (dry AMD)	0.31 (0.26 to 0.43)	0.25 (0.14 to 0.33)	0.41 (0.31 to 0.50)	0.39 (0.36 to 0.54)	0.42 (0.36 to 0.54)	0.43 (0.37 to 0.56)
Area under ROC (dry AMD)	0.61 (0.53 to 0.71)	0.67 (0.63 to 0.71)	0.67 (0.58 to 0.76)	0.66 (0.58 to 0.76)	0.68 (0.70 to 0.78)	0.69 (0.61 to 0.78)
Sensitivity (wet AMD)	60.67% (54.89% to 66.24%)	71.06% (66.02% to 76.79%)	71.21% (65.01% to 74.91%)	64.52% (57.68% to 76.03%)	66.03% (67.00% to 76.41%)	72.21% (66.3% to 78.41%)
Specificity (wet AMD)	63.76% (56.40% to 69.54%)	54.18% (47.05% to 61.29%)	66.36% (61.71% to 74.99%)	60.19% (55.18% to 78.48%)	62.66% (64.62% to 77.99%)	67.03% (62.18% to 73.48%)
Accuracy (wet AMD)	62.28% (58.67% to 66.63%)	63.05% (58.39% to 67.59%)	63.44% (60.89% to 70.77%)	62.41% (67.02% to 75.96%)	65.01% (67.86% to 75.56%)	69.93% (67.99% to 73.71%)
Unweighted kappa (wet AMD)	0.32 (0.27 to 0.39)	0.25 (0.15 to 0.32)	0.39 (0.30 to 0.48)	0.42 (0.36 to 0.53)	0.42 (0.36 to 0.53)	0.43 (0.36 to 0.53)
Area under ROC (wet AMD)	0.59 (0.55 to 0.64)	0.66 (0.63 to 0.71)	0.63 (0.59 to 0.77)	0.65 (0.52 to 0.80)	0.68 (0.62 to 0.80)	0.69 (0.62 to 0.80)

## Data Availability

The training data are available in the public domain upon request from dbGaP that holds the AREDS image and genotype datasets. The code and trained models are available upon request.

## Appendix A

**Table A1:** Predictive values and beta coefficients of the SNPs included in our AMD disease prediction model.

		Any Late AMD		Dry AMD (Geographic Atrophy)		Wet AMD (Neovascular AMD)	
SNP/Allele	Gene	Beta Coefficient	*p*-Value	Beta Coefficient	*p*-Value	Beta Coefficient	*p*-Value
rs572515 GG	CFH	0	n/a	0	n/a	0	n/a
rs572515 AA	CFH	0.2476	0.551	0.3319	0.6016	−0.2892	0.5372
rs572515 AG	CFH	0.1399	0.6903	0.0637	0.9034	−0.146	0.7152
rs380390 CG	CFH	0	n/a	0	n/a	0	n/a
rs380390 CC	CFH	−0.3697	0.3883	0.0346	0.9545	−0.7816	0.13
rs380390 GG	CFH	−0.1313	0.6557	−0.1149	0.8029	0.132	0.6966
rs1329428 CT	CFH	0	n/a	0	n/a	0	n/a
rs1329428 TT	CFH	0.5493	0.1197	−0.2292	0.6785	0.7087	0.1101
rs1329428 CC	CFH	0.6058	0.0011	0.0862	0.7431	0.6998	0.0035
rs10801575 CT	CFH	0	n/a	0	n/a	0	n/a
rs10801575 CC	CFH	0.1195	0.527	0.0076	0.9782	0.1903	0.4189
rs10801575 TT	CFH	−0.7079	0.054	−0.4492	0.3564	−0.514	0.2722
rs800292 AG	CFH	0	n/a	0	n/a	0	n/a
rs800292 GG	CFH	−0.0033	0.9884	0.2942	0.3734	−0.2499	0.389
rs800292 AA	CFH	0.1808	0.6951	−0.9109	0.3995	0.6387	0.203
rs10490924 GT	ARMS2	0	n/a	0	n/a	0	n/a
rs10490924 GG	ARMS2	−0.7436	0	−0.3108	0.1094	−0.8845	0
rs10490924 TT	ARMS2	0.3716	0.0189	0.2648	0.2702	0.3332	0.0691

**Table A2:** The short list of significant genes which are associated to the incidence of late AMD are shown here. The cumulative data of significance of genes related to AMD which are selected in this study based on existing research. ARMS2 and CFH were consistently found in multiple research publications. Other genes found to be associated with different types of AMD with varying levels are also shown concisely in the table.

Type of Disease/Association	Genes/SNPs
Wet AMD	ARMS2, CFH, C3, LOXL1, HTRA1, C2-rs547154, ABCA1
Dry AMD	CFH, SELP
AMD (general)	CFB, FBLN5, SERPING1, Tf (smoker), CACNG3, C9, CFI
Possible link to AMD	ERCC6
Reduced Risk	LIPC
